# Bis(dimethylpyrazolyl)-aniline-*s*-triazine derivatives as efficient corrosion inhibitors for C-steel and computational studies

**DOI:** 10.1098/rsos.231229

**Published:** 2024-05-08

**Authors:** Hassan H. Hammud, Nadeem S. Sheikh, Ihab Shawish, Hawra A. Bukhamsin, Dolayl E. Al-Hudairi, Angelina L. X. Wee, Malai Haniti S. A. Hamid, Sarah A. Maache, Hessa H. Al-Rasheed, Assem Barakat, Ayman El-Faham, Hany M. Abd El-Lateef

**Affiliations:** ^1^ Department of Chemistry, College of Science, King Faisal University, Al-Ahsa, 31982, Saudi Arabia; ^2^ Chemical Sciences, Faculty of Science, Universiti Brunei Darussalam, Gadong BE1410, Brunei; ^3^ Department of Math and Sciences, College of Humanities and Sciences, Prince Sultan University, Riyadh, 11586, Saudi Arabia; ^4^ Leading National Academy, Khobar Niagara College, Al Khobar, Saudi Arabia; ^5^ Department of Chemistry, College of Science, King Saud University, Riyadh, 11451, Saudi Arabia; ^6^ Chemistry Department, Faculty of Science, Alexandria University, Alexandria, 21321, Egypt

**Keywords:** 1,3,5-triazines, C-steel, electrochemical studies, anti-corrosion, density functional theory calculations, Monte Carlo simulation

## Abstract

4,6-Bis(3,5-dimethyl-*1H*-pyrazol-1-yl)-*N*-phenyl-1,3,5-triazin-2-amine (**PTA-1**), *N*-(4-bromophenyl)-4,6-bis(3,5-dimethyl-*1H*-pyrazol-1-yl)-1,3,5-triazin-2-amine (**PTA-2**) and 4,6-bis(3,5-dimethyl-*1H*-pyrazol-1-yl)-*N*-(4-methoxyphenyl)-1,3,5-triazin-2-amine (**PTA-3**) were synthesized and characterized. Their corrosion inhibition of carbon C-steel in 0.25 M H_2_SO_4_ was studied by electrochemical impedance. The inhibition efficiency (IE%) of triazine was superior due to the cumulative inhibition of triazine core structure and pyrazole motif. Potentiodynamic polarizations suggested that *s*-triazine derivatives behave as mixed type inhibitors. The IE% values were 96.5% and 93.4% at 120 ppm for inhibitor **PTA-2** and **PTA-3** bearing –Br and –OCH_3_ groups on aniline, respectively. While **PTA-1** without an electron donating group showed only 79.0% inhibition at 175 ppm. The adsorption of triazine derivatives followed Langmuir and Frumkin models. The values of adsorption equilibrium constant *K*°_ads_ and free energy change Δ*G*°_ads_ revealed that adsorption of inhibitor onto steel surface was favoured. A corrosion inhibition mechanism was proposed suggesting the presence of physical and chemical interactions. Density functional theory computational investigation corroborated nicely with the experimental results. Monte Carlo simulation revealed that the energy associated with the metal/adsorbate arrangement d*E*_ads_/d*N*_i_, for both forms of **PTA-2** and **PTA-3** with electron donating groups (−439.73 and −436.62 kcal mol^−1^) is higher than that of **PTA-1** molecule (−428.73 kcal mol^−1^). This aligned with experimental inhibition efficiency results.

## 1. Introduction

Owing to their strength and cost effectiveness, iron alloys are undoubtedly considered as the efficient constructive backbones of metallic structures [1]. Due to its widespread applications, iron steel is being used in almost every aspect of our daily lives and, consequently, is subjected to constant modifications to enhance structural features [2,3].

Corrosion of iron-containing metals, including carbon steel, cast iron and other alloys, poses a major challenge that can lead to significant metallic losses and substantial reduction in their mechanical properties [4]. The structural changes caused by corrosion is due to the degradation of iron content and the formation of brittle layers of rust. It is estimated that corrosion can lead to the loss of approximately 10% of the world’s total metal output [5–7]. A common economic impact of corrosion on industries is related to the damaged facilities which may require the maintenance, repair or replacement of corroded equipment. Nevertheless, certain applications can avoid leakages or fractures that may lead to fatalities and are of serious environmental concerns [8–11].

Carbon steel is one of the readily available, low-cost iron alloys that contains up to 1% carbon. It is primarily used in the oil and gas industry to manufacture machines, wire ropes, seawater petroleum pipelines and other industrial appliances [11]. However, carbon steel is highly vulnerable to corrosion and considered unstable under acidic conditions which causes major metallic destruction, leading to tremendous economic losses.

**Figure 1.**
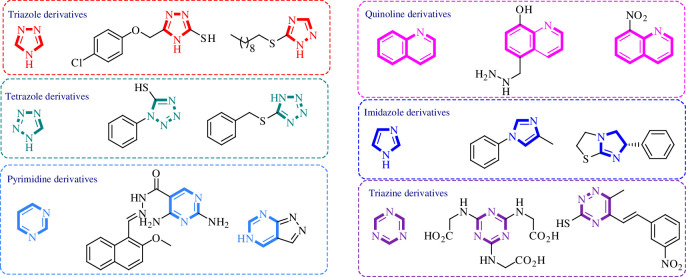
Representative examples illustrating application of heterocyclic organic molecules as efficient corrosion inhibitors.

Corrosion prevention methods are specific protocols and strategies that are associated with decreasing the losses and damages caused by corrosion on industrial scale [12,13]. Each method depends on several factors including the type of corrosion, metal surface, pH of the medium, temperature and other relevant aspects. Accordingly, several protocols have been reported to mitigate the harmful impact of corrosion including design modification [14], sacrificial anodes [15,16] and surface coating [17–19]. The latter method relies on the formation of a protective layer using a proper material that suits both the type of metal and the environmental conditions to prevent the phenomenon of corrosion. The mechanism of this strategy is based on the adsorption of the protective material on the surface of the metal to form a protective layer. Several approaches highlighted the application of nanocomposites [20], inorganic compounds [21,22] and natural extracts [23] as cheap corrosion inhibitors with diversified applications and high durability on the metallic surface. At the same time, several studies reported toxicity concerns regarding the high concentrations of some inhibition agents such as hexavalent chromium [24,25] and mercapto-benzothiazole derivatives [26]. In addition, structurally diversified heterocyclic organic molecules have also been explored as efficient corrosion inhibition agents (figure 1). Due to the presence of π-bonds and heteroatoms such as nitrogen (N), oxygen (O) and sulfur (S), these compounds exhibit excellent corrosion inhibition profiles because the heteroatoms are known to ease the adsorption process on the metal surface which assists in protection of the metallic surface from corrosion. There is a large number of heterocyclic structures which have been reported as corrosion inhibitors including triazoles [27], tetrazoles [28], pyrimidines [29], quinolones [30], imidazoles [31], triazines [32], isoxazoles [33], pyrazoles [34], pyridines [35], pyrazines [36], pyridazines [37] and many more.

The new *s*-triazines prepared are expected to provide an anticorrosion coat for steel and iron in analogy with other related organic inhibitors, which when mixed with other ingredients provide uniform coating, dried surface and being less permeable to water and oxygen. The applied coatings can have a lifetime of up to 10 years [38].

Moreover, density functional theory (DFT) has been applied to predict the corrosion inhibition efficiency of organic molecules and to design efficient corrosion inhibitors [39].

**Figure 2.**
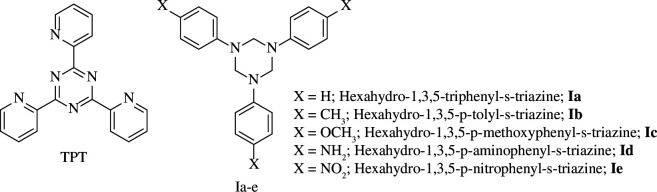
Tripyridyl and triphenyl *s*-triazine derivatives as corrosion inhibitors [40,41].

Recently, Xuehui *et al*. [40] described the inhibitive effect of 2,4,6-tri(2-pyridyl)-*s*-triazine (**TPT**, figure 2) on the corrosion of steel in 1 M hydrochloric acid (HCl). Scanning electron microscopy (SEM) analysis indicated that the metal was protected from destructive corrosion by the addition **TPT**.

More recently, five prepared triazines **Ia–e** were reported as corrosion inhibitors of steel in 1 N HCl solution. The inhibitory action of the triazines depended on the type of electronic groups present (figure 2) [41].

**Figure 3.**
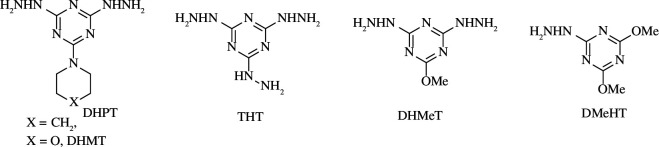
Hydrazino-*s*-triazine derivatives as corrosion inhibitors [42–44].

Very recently, the El-Faham group [42–44] reported several hydrazino-*s*-triazine derivatives (figure 3). They suggested that the number of hydrazino groups plays a vital role in the inhibition of corrosion. Additionally, the presence of oxygen as in 2,4-dihydrazino-6-morpholino-1,3,5-triazine (DHMT), 2,4-dihydrazino-6-methoxy-1,3,5-triazine (DHMeT) and 2-hydrazino-4,6-dimethoxy-1,3,5-triazine (DMeHT) renders the compounds more protected due to the formed film on the steel surface through lone pair sharing.

**Figure 4.**
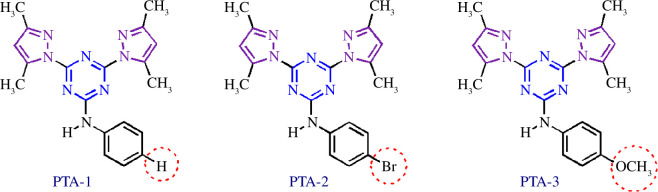
Structures of organic molecules synthesized and explored for application as corrosion inhibitors in this study.

Owing to significant interests of scientific community in preparing efficient organic corrosion inhibitors, we reported a facile synthesis of novel tri-substituted 1,3,5-triazine containing pyrazole ring and aromatic amines (figure 4) [45]. The synthesized compounds were subjected to detailed synergistic experimental and computational studies to evaluate their potential application as efficient corrosion inhibitors for carbon steel in an acidic medium. Thus, the newly prepared structures 4,6-bis(3,5-dimethyl-1*H*-pyrazol-1-yl)-*N*-phenyl-1,3,5-triazin-2-amine (PTA-1), *N*-(4-bromophenyl)-4,6-bis(3,5-dimethyl-1*H*-pyrazol-1-yl)-1,3,5-triazin-2-amine (PTA-2) and 4,6-bis(3,5-dimethyl-1*H*-pyrazol-1-yl)-*N*-(4-methoxyphenyl)-1,3,5-triazin-2-amine (PTA-3) were synthesized. They contained three inhibition moieties that can have cumulative anticorrosion effects: aniline, pyrazole and triazine containing one N, two N and three N sigma electrons donor atoms, respectively, and π aromatic rings. This novel strategy makes the new compounds possess superior anticorrosion properties at low concentrations compared to other compounds reported in the literature. The PTA compounds’ inhibition reached ~95% at 120 ppm concentration, while other reported compounds in the literature required 250–5000 ppm to attain inhibition greater than ~95%. In addition, the aniline moiety has different *para*-substituents, namely hydrogen, bromine and methoxy (–H, –Br, –OMe), in order to explore the effect of substituent on inhibition.

Several recent reports have demonstrated the significance of computational calculations for evaluating the inhibition efficiency of organic corrosion inhibitors [46–50]. In addition, mechanisms were postulated for the physio-chemical adsorption of heterocyclic structures on the surface of metals. However, advanced progress in the topic adopted Monte Carlo (MC) simulations to provide more insight into the interactions between the corrosion inhibitors and the metal surface [51,52].

## 2. Experimental

### 2.1. General procedure for the synthesis of bispyrazolyl-*s*-triazine derivatives PTA-1–3

Monopyrazolyl-*s*-triazine derivatives **PTA-1**–**PTA-3** synthesized by the reaction of the hydrazine-*s*-triazine derivatives with acetylacetone in the presence of triethylamine base gave the desired pure products in high yield by following the reported method in the literature [45] (electronic supplementary material, synthetic method).

### 2.2. Electrochemical measurements

The electrochemical cell used in this study consists of three electrodes. Platinum (Pt) wire was used as counter electrode. The reference electrode used was a saturated silver/silver chloride (Ag/AgCl) electrode. The working electrode consisted of carbon steel rod with the following chemical composition (wt%): carbon (C), 0.164; sulfur (S), 0.001; manganese (Mn), 0.710; phosphorus (P), 0.0005; silicon (Si), 0.26; nickel (Ni), 0.123; chromium (Cr), 0.041; and iron (Fe), balance; the exposed area of the cylindrical C-steel rod is 0.5027 cm^2^. The choice of substrate C-steel is because it is used in industrial oil and water pipes. The choice of corrosive medium 0.25 M H_2_SO_4_ is because this acid is used to wash pipes from build-up of undesirable coat, and eventually causing corrosion of the pipes. The test solution used in all experiments was 0.25 M H_2_SO_4_ containing different concentrations of the triazine inhibitors (10, 25, 40, 50, 60, 100, 120, 150 and 175 ppm) [23]. The compounds PTA-1, PTA-2 and PTA-3 are soluble at the concentrations used in 0.25 M H_2_SO_4_. In general, PTA-2 and PTA-3 are more soluble than PTA-1, because PTA-2 and PTA-3 contain polar groups –Br and –OCH_3_.

Potentiodynamic polarization (PDP) study and electrochemical impedance spectroscopy (EIS) were conducted with a Gamry reference 600 Potentiostat/Galvanostat/ZRA (Warminster, PA) using Gamry software v. 7.07. At the beginning of the experiment, the steady-state open circuit potential EOCP was recorded by immersing the electrode in the corrosion solution for 1 h until reaching a steady state, with the indication 10 mV disturbance capacity [23]. EIS was conducted in the frequency range 0.1–100 000 Hz with a signal amplitude perturbation of 10 mV around the corrosion potential [42–44]. The polarization curves were recorded with a sweep rate of 5 mV s^−1^, by automatically polarizing the working electrode from 500 mV versus the rest potential [43].

### 2.3. Computational methods

DFT [53] studies were performed using Gaussian 16 (revision C.01) [54] and Gaussview 6.1.1 [55] was used to generate input geometries and for visualization of the output structures. Geometry optimizations and frequency calculations for the heterocyclic structures PTA-1, PTA-2 and PTA-3 neutral and protonated forms were carried out using the B3LYP functional [56–58] with the 6-311++G(d,p) basis set [59]. Electronic and structural parameters, electronic energies and dipole moment values were obtained from the optimization and frequency calculations. The vertical ionization potentials (*I*) and electron affinities (*A*) were calculated by obtaining the excitation energetics of the neutral, radical cation and radical anion species. This approach based on excitation energetics is considered more accurate compared to generally adopted Koopmans’ theorem which approximates (*I*) and (*A*) by equating to the energy values of highest occupied molecular orbital (HOMO) and lowest unoccupied molecular orbital (LUMO), respectively. As the latter approach is generally accepted, a comparative study has also been conducted. Quantum chemical parameters were calculated from the obtained values of (*I*) and (*A*), which have been extensively studied to rationalize the corrosion inhibition efficiency of organic inhibitors [60]. The calculations were initially performed in the gaseous phase; however, for comparison and to model the solvation effect, the optimization and frequency calculations were carried out by employing the integral equation formalism (IEF) version of polarized continuum model (PCM) solvation model for water (relative permittivity ε = 78.4) [61,62]. This is crucial as the corrosion process occurs in an aqueous phase. All stationary points were characterized as minima based on normal vibrational mode analysis. Thermal corrections were calculated from unscaled frequencies, assuming a standard state of 298.15 K and 1 atm. The structures presented here are the lowest energy-optimized conformers.

### 2.4. Monte Carlo simulations

The proper adsorption arrangements of the PTA1, PTA2 and PTA 3 molecules on the Fe(110) surface were determined using the adsorption locator module in the Materials Studio v. 7.0 program [63]. Initially, the adsorbate molecules were optimized using the COMPASS force field [64]. Subsequently, the adsorption of the investigated inhibitors, including Cl^−^ ions, hydronium ions and water molecules, with the Fe(110) surface was simulated within a simulation box measuring 37.24 × 37.24 × 59.81 Å^3^ [65].

## 3. Results and discussion

### 3.1. Synthesis of inhibitors

**Scheme 1.**
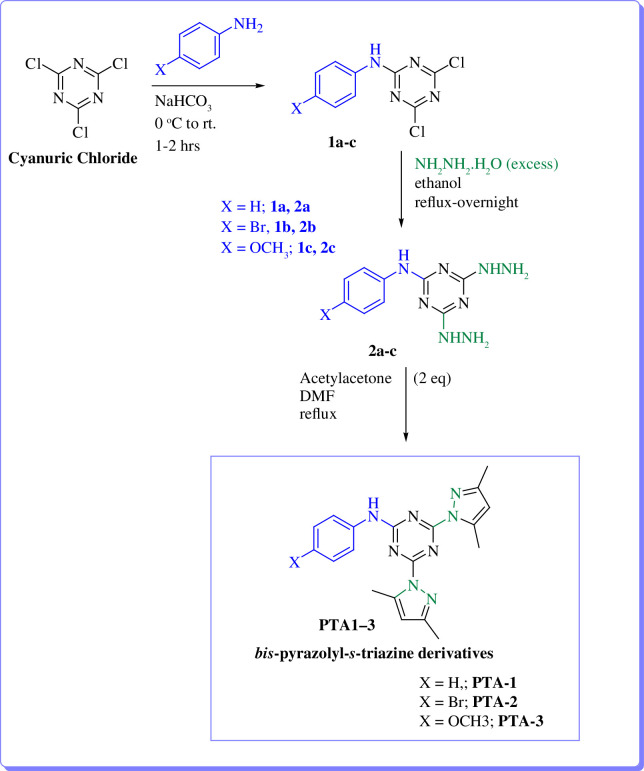
Synthesis pathway of *s*-triazine derives **PTA-1**–**PTA-3** [45].

To explore further the application of the *s*-triazine-based molecules as corrosion inhibitors, three compounds having different electronic effects with either electron donating or withdrawing effects were selected, resynthesized and characterized by the El-Faham group [45]. The target compounds were prepared according to scheme 1 [45,66].

The structures of the three derivatives PTA-1–PTA-3 were confirmed by NMR and agreed with the reported data [45] (electronic supplementary material, synthetic method).

FTIR analysis was conducted with a Shimadzu 8300 FTIR spectrophotometer between 500 and 4000 cm^−1^ in order to characterize the three Scheme 1compounds. Their FTIR spectra are shown in electronic supplementary material, figure S1. The FTIR absorption peak wavenumbers (cm^−1^) were observed for **PTA-1**: 3256.2, 3001.6, 29369.9, 1601.1, 1578.0, 1551.0, 1489.2, 1404.4, 1350.3, 1142.0, 1045.5, 987.7, 817.9, 760.1, 702.2, 698.3, 582.6; for **PTA-2**: 3306.4, 3024.8, 2963.0, 1597.3, 1547.1, 1396.6, 1385.1, 1138.1, 1095.7, 1041.7, 980.0, 814.1 756.2, 663.6, 586.4; and for **PTA-3**: 3206.1, 3115.9, 2997.7, 2932.2, 2835.7, 1593.4, 1512.4, 1408.2. 1365.8, 1230.7, 1176.7, 1130.4, 1037.8, 976.1, 806.3, 744.6, 559.4.

### 3.2. Electrochemical impedance studies

The open circuit potential (OCP) was measured in the blank (0.25 M H_2_O_4_) and in the presence of inhibitors **PTA-1**, **PTA-2** and **PTA-3**. The system could reach almost steady state after 1 h of OCP test. Therefore, 1 h is chosen as stabilization time for further EIS and polarization measurements. As the concentration of **PTA-1** increased, the values of OCP are changed toward more positive values compared with the OCP value in the blank. This can be explained by the formation of an inhibition film on the carbon steel surface (electronic supplementary material, figure S2*a*) [67]. While there was no obvious trend in the case of **PTA-2** and **PTA-3** (electronic supplementary material figure S2*b*,*c*). In the case of **PTA-3**, the OCP values were more positive than that of the blank up to 1000 s, then they shifted to more negative values towards more cathodic ones. Similar trend was observed in the case of **PTA-2** with some exceptions [68].

EIS data were fitted by electrical equivalent circuits (EECs) model 1, 2 and 3.

**Figure 5.**
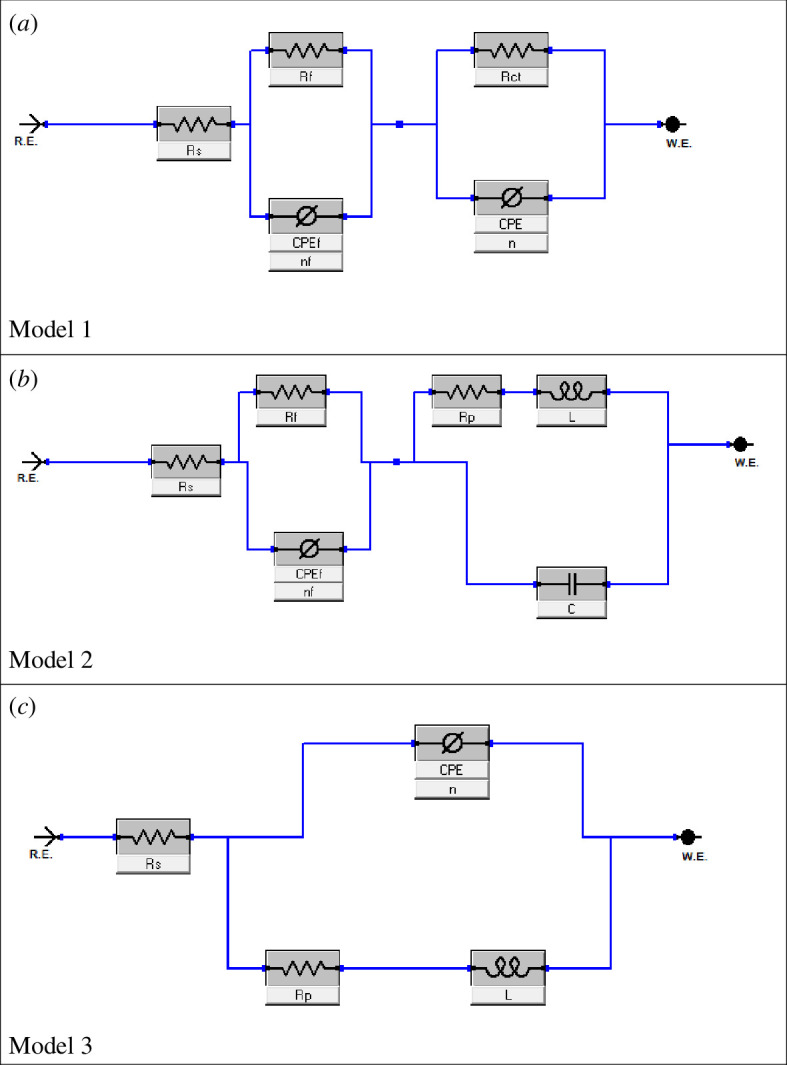
Equivalent circuits (*a*) model 1, (*b*) model 2 and (*c*) model 3 used to fit the impedance data.

The EEC model 1 comprised a solution resistance (*R*_s_) and two circuits (time constants) connected in series. The first time constant consisted of a film resistance (*R*_f_) in parallel with film constant phase element (CPE_f_, figure 5*a*) [44]. While the second time constant contains a charge transfer resistance (*R*_ct_) in parallel with a constant phase element of double-layer CPE_dl_ as shown in figure 5*a*. The double-layer capacitance (*C*_dl_) can be computed from *R*_ct_, the impedance of CPE_dl_ (*Z*_CPE_) and the exponents of CPE_dl_ (*n*) [69].

EEC model 2 (figure 5*b*) is like EEC model 1, but a pure double layer capacitor (*C*_dl_) replaced the constant phase element (CPE_dl_) and polarization resistance *R*_p_ relaced *R*_ct_ of model 1. Where *R*_p_ has two contributions *R*_ct_ and inductor resistance *R*_L_. Additionally, an inductance *L* component was added in model 2 [70] in order to account for the small inducive loop at low frequencies. The inductance *L* is the tendency of an electric conductor to oppose a change in the electric current flowing through it. The SI unit of inductance is henry abbreviated as H.

EEC model 3 (figure 5*c*) comprised a solution resistance (*R*_s_) and one circuit (time constant). The circuit consisted of a constant phase element (CPE), a resistance (*R*_p_) and an inductance element (*L*); *R*_p_ having contribution from both film resistance (*R*_f_) and charge transfer resistance (*R*_ct_).

**Figure 6.**
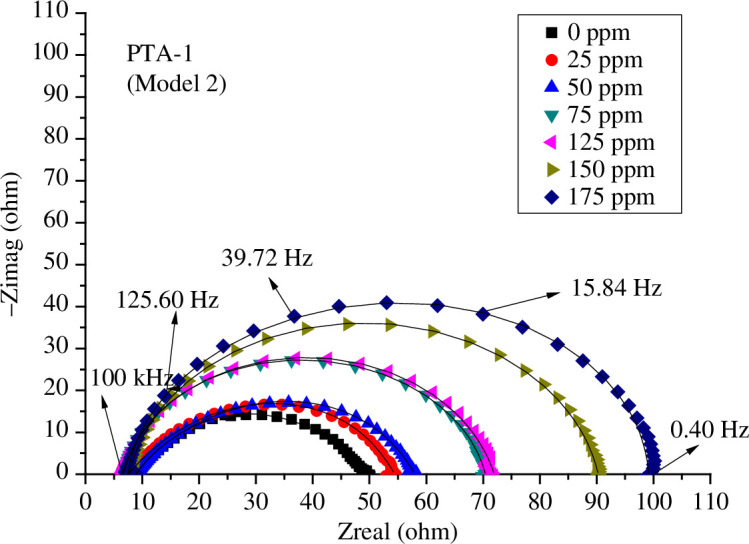
Nyquist plots of *s*-triazine (**PTA-1**) at different concentrations and 298 K in 0.25 M H_2_SO_4_ fitted by model 2.

**Figure 7.**
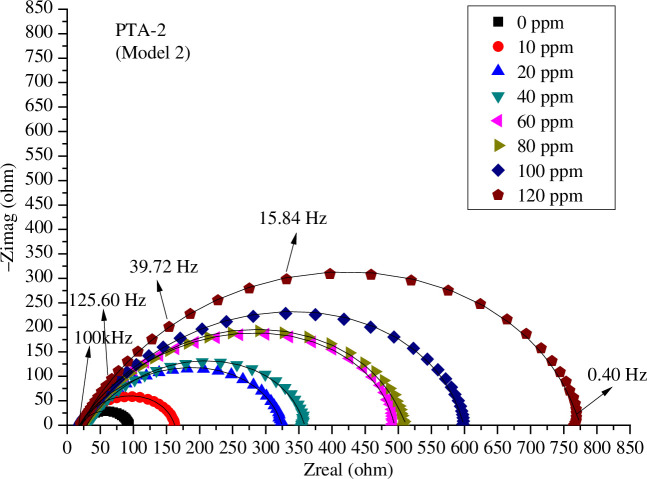
Nyquist plots for *s*-triazine (**PTA-2**) at different concentrations and 298 K in 0.25 M H_2_SO_4_ fitted by model 2.

**Figure 8.**
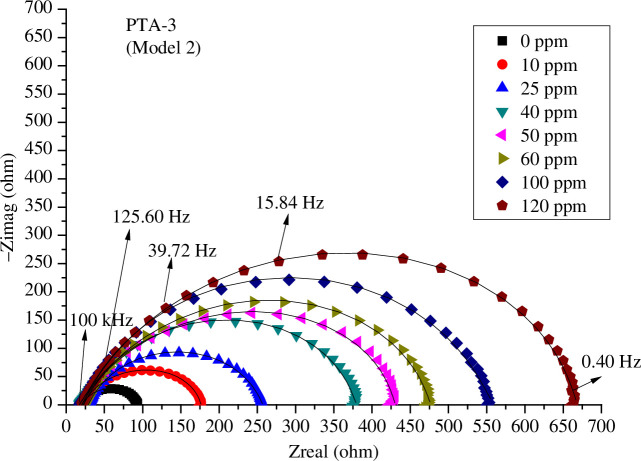
Nyquist plots for *s*-triazine (**PTA-3**) at different concentrations and 298 K in 0.25 M H_2_SO_4_ fitted by model 2.

The influence of concentrations of **PTA-1**, **PTA-2** and **PTA-3** on the Nyquist plots of C-steel in 0.25 M sulfuric acid solution is shown, respectively, in figures 6–8 using model 2; and electronic supplementary material, figure S3*a*–*c*, using model 1, and figure S3*d*–*f*, using model 3. It is obvious that the higher the concentration of **PTA-1**, **PTA-2** and **PTA-3**, the larger the size of the capacitive loops.

It should be noted that the fitting by EEC model 3 using one time constant was not good since it deviated well from the experimental value (electronic supplementary material, figure S3*d*–*f*). That is why we have used two time constants in model 1 and model 2 [23,35]. Model 2 has the advantage of accounting for the inductance loop; the fitting of Nyquist plots was also perfect (figures 6–8). In general, every semicircle in the Nyquist plot should be represented by one time constant, but it happened as it is in the present case, two time constants show only one major semicircle in the Nyquist plot. The first semicircle can be very small at high frequency and merged with the larger semicircle as was found in other studies. Thus, the first semicircle was not observed due to the high-frequency limit [35].

All the measured impedance parameters are reported in tables 1–3 by using model 2 and electronic supplementary material, tables S1–S3, by using model 1 for **PTA-1**, **PTA-2** and **PTA-3**, respectively. The inhibition efficiency %IE = 100 × Ɵ and the surface coverage Ɵ = 1 – [*R*_(0)_/*R*_(i)_] were calculated; where *R* = *R*_ct_ + *R*_f_ for model 1 and *R* = *R*_p_ + *R*_f_ for model 2 [23].

**Table 1.** Impedance parameters for **PTA-1** on C-steel in 0.25 M sulfuric acid by fitting the equivalent circuit model 2 (± error).

PTA-1	*R*_s_	*R*_f_	*n*_f_	*Z*_CPEf_	*R*_p_	*C*_dl_	*R*_f_ + *R*_p_	*θ*	IE%	*L*
ppm	Ω cm^2^ ± 0.33	Ω cm^2^ ± 1.88	± 0.13	µΩ^−1^s^*n*^ cm^−2^ ± 21.71	Ω cm^2^ ± 2.22	μF cm^−2^ ± 11.71	Ω cm^2^ ± 4.1		± 0.60	H
0	4.50	19.62	0.709	1024	0.98	3266	20.60	0.0000	0.00	2.88E-07
25	3.86	15.18	0.677	1679	8.36	1121	23.54	0.1248	12.48	1.64E-02
50	4.45	16.89	0.686	1351	7.83	953	24.72	0.1668	16.68	1.31E-05
75	3.36	22.44	0.860	450	9.60	391	32.04	0.3570	35.70	1.02E-05
125	3.24	19.36	0.808	641	13.58	350	32.94	0.3747	37.47	1.38E-02
150	3.85	27.19	0.859	336	14.35	354	41.53	0.5040	50.40	1.63E-02
175	3.77	20.35	0.917	250	25.71	269	46.06	0.5528	55.28	2.03E-03

**Table 2.** Impedance parameters for **PTA-2** on C-steel in 0.25 M sulfuric acid by fitting the equivalent circuit model 2 (± error).

PTA-2	*R*_s_	*R*_f_	*n*_f_	*Z*_CPEf_	*R*_p_	*C*_dl_	*R*_f_+ *R*_p_	*θ*	IE%	*L*
ppm	Ω cm^2^ ± 0.48	Ω cm^2^ ± 3.64	± 0.035	µΩ^−1^ s^*n*^ cm^−2^ ± 12.22	Ω cm^2^ ± 4.10	µF cm^−2^ ± 7.4	Ω cm^2^ ± 7.74		± 0.55	H
0	10.29	19	0.748	855	17.42	431	36.42	0.0000	0.00	2.67E-06
10	9.43	28.54	0.687	798	43.83	203	72.37	0.4968	49.68	7.63E-02
20	9.53	83.8	0.731	256	70.03	201	153.83	0.7632	76.32	1.62E-01
40	14.95	77.16	0.721	281	88.32	147	165.49	0.7799	77.99	1.77E-01
60	10.58	111.75	0.761	158	126.28	111	238.03	0.847	84.70	2.49E-04
80	10.81	109.54	0.671	314	137.59	98	247.13	0.8526	85.26	2.37E-03
100	9.28	129.85	0.716	185	162.32	107	292.17	0.8753	87.53	5.66E-01
120	10.17	160.31	0.715	197	219.58	74	379.89	0.9041	90.41	4.53E-01

**Table 3.** Impedance parameters for **PTA-3** extract on C-steel in 0.25 M sulfuric acid by fitting the equivalent circuit model 2 (± error).

PTA-3	*R*_s_	*R*_f_	*n*_f_	*Z*_CPEf_	*R*_p_	*C*_dl_	*R*_f_ + *R*_p_	*θ*	IE%	*L*
ppm	Ω cm^2^ ± 0.41	Ω cm^2^ ± 4.75	± 0.05	µΩ^−1^ s^*n*^ cm^−2^ ± 11	Ω cm^2^ ± 4.64	µF cm^−2^ ± 5.5	Ω cm^2^ ± 11.55		± 0.50	H
0	10.29	19	0.748	855	17.42	431	36.42	0.00	0.00	2.67E-06
10	11.36	48.09	0.803	272	29.26	359	77.36	0.53	52.91	6.37E-02
25	14.44	61.38	0.742	388	53.54	169	114.92	0.68	68.30	9.18E-02
40	8.56	74.7	0.698	395	107.93	129	182.63	0.80	80.05	2.52E-01
50	10.33	99.94	0.754	182	106.27	122	206.21	0.82	82.33	2.05E-01
60	11.34	114.46	0.749	207	114.01	123	228.48	0.84	84.06	2.55E-01
100	8.64	111.8	0.674	491	160.76	109	272.56	0.87	86.63	3.75E-01
120	10.28	148.9	0.754	180	177.1	103	326	0.89	88.83	5.27E-01

The Nyquist diagrams are composed of a small inducive loop at low frequencies and a great capacitive loop at high frequencies. The capacitive loop increased with increasing inhibitor concentrations. This indicated that the corrosion inhibition is governed by charge transfer [63].

It is noted that *R*_f_ + *R*_ct_ values measured by model 1 (electronic supplementary material, tables S1–S3) and *R*_f_ + *R*_p_ values by model 2 (tables 1–3) increased with increase in the amount of inhibitors **PTA-1**, **PTA-2** and **PTA-3**, respectively. Also, the surface coverage of steel Ɵ increased with higher concentration due to greater amount of adsorbed **PTA-1**, **PTA-2** and **PTA-3** on the steel surface, causing higher IE% [43]. **PTA-2** and **PTA-3** having electron rich groups –Br and –CH_3_ showed a maximum inhibition value IE% computed from both models 1 and 2, 90.4% and 88.83%, respectively, at the concentration 120 ppm (electronic supplementary material, tables S2 and S3; tables 3 and 4). While **PTA-1** has 57.9% at 175 ppm (electronic supplementary material, table S1; table 1). The compounds **PTA-1**, **PTA-2** and **PTA-3** are soluble at the concentrations studied. The solubility increased in the following order: **PTA-1** < **PTA-3** < **PTA-2**. Thus, this electrochemical study could not cover inhibition at wider concentration range; at higher concentration, there was no improvement in % inhibition. Additionally, precipitation of excess insoluble amounts at higher concentrations occurred in the case of inhibitor **PTA-3**. Also, the semicircles of Nyquist plots obtained by impedance at higher concentrations than the optimum concentration of 120 ppm for **PTA-2** and **PTA-3**, and 175 ppm for **PTA-1** became depressed compared to the respective optimum concentrations. Thus the % inhibition values decreased instead of increasing above the optimum concentrations [62]. The CPE frequency exponent (*n*) values are reported in electronic supplementary material, tables S1–S3, when using EEC model 1 for **PTA-1**, **PTA-2** and **PTA-3**. The increase in the *n* value to about 0.9 in the case of the inhibited system, compared to the *n* value of about 0.7 obtained in the blank acidic medium, could be related to a lessening of surface heterogeneities.

**Table 4.** Phase angles and *α* values for **PTA-1**, **PTA-2** and **PTA-3** at various concentrations.

		0 ppm	25 ppm	50 ppm	75 ppm	125 ppm	150 ppm	175 ppm	
**PTA-1**	phase angle (°)	−30.57	−33.39	−31.84	−49.11	−49.11	−51	−53.79	
	frequency (Hz)	79.03	79.03	79.03	125.80	125.80	125.80	125.80	
	slope *α*	−0.3286	−0.3823	−0.3765	−0.5965	−0.5917	−0.6192	−0.65373	
	*R* ^2^	0.9844	0.9915	0.9946	0.995	0.9953	0.9951	0.99625	

		0 ppm	10 ppm	20 ppm	40 ppm	60 ppm	80 ppm	100 ppm	120 ppm
**PTA-2**	phase angle (°)	−30.92	−41.37	44.79	−42.89	−49.57	−48.31	−50.3	−54.89
	frequency (Hz)	63.34	63.34	63.34	63.34	79.00	79.00	79.00	79.00
	slope *α*	−0.3653	−0.4952	−0.5493	−0.5194	−0.5988	−0.5784	−0.59905	−0.6588
	*R* ^2^	0.9879	0.9928	0.9991	0.9976	0.9993	0.9991	0.99971	0.99971

		0 ppm	10 ppm	25 ppm	40 ppm	50 ppm	60 ppm	100 ppm	120 ppm
**PTA-3**	phase angle (°)	−30.57	−39.8	−42.83	−48	−49.38	−50.8	−54.5	−55.12
	frequency (Hz)	79.03	79.03	79.03	79.03	79.03	79.03	79.03	79.03
	slope *α*	−0.3279	−0.4463	−0.47	−0.5764	−0.5717	−0.5717	−0.62133	−0.6341
	*R* ^2^	0.9766	0.9901	0.9852	0.9954	0.9974	0.9965	0.99332	0.99771

The similarity in semicircle shapes of the Nyquist plots at different concentrations revealed that the corrosion inhibition process occurred with a charge transfer mechanism [40].

The constant phase element of double layer impedance of CPE_dl_ (*Z*_CPE_) was also computed when using EEC model 1. The values of *Z*_CPE_ (µΩ^−1^ s^*n*^ cm^−2^) are presented in electronic supplementary material, tables S1–S3. In the case of **PTA-2**, the values decreased from 2740.1 for the blank to 358.2 for solution with concentration 120 ppm (electronic supplementary material, table S2). Also, for **PTA-3**, the values decreased to 322.3 for 120 ppm solution (electronic supplementary material, table S3) indicating more corrosion inhibition.

The double layer capacitance *C*_dl_ was computed directly by fitting EEC model 2 using Gamry software v.7 (tables 1–3). However, when fitting data by EEC model 1, the *C*_dl_ values were computed by Gamry software using *R*_p_, *n* and *Z*_CPE_ values. *C*_dl_ can be also calculated using Brug’s formula [71]

*C*_dl_ values decrease with the increase in **PTA-1**, **PTA-2** and **PTA-3** concentrations. This is due to the replacement of water molecules by adsorbed molecules of lower dielectric constant. Thus, the greater the concentration of inhibitors **PTA-1**, **PTA-2** and **PTA-3**, the greater the thickness of the adsorbed layer, and the smaller is the electrical capacitance [72]. Both results of increasing *R*_f_ + *R*_ct_ (model 1) and *R*_f_ + *R*_p_ (model 2), and decreasing *C*_dl_ values suggest that the adsorbed **PTA-1**, **PTA-2** and **PTA-3** block the active sites on the metal surface and formed a protective coat [28].

The inductive loop in Nyquist plots (figures 6–8) can be attributed to adsorption of ions, neutral molecules or insoluble corrosive products, but may be also due to aggressive sulfate ions, which produce instability of electrode surface [70]. The inductance *L* values increased with increasing concentrations of inhibitors **PTA-1**, **PTA-2** and **PTA-3** (tables 1–3).

The film double layer capacitance *C*_dlf_ was also calculated similarly as above form film resistance *R*_f_, film CPE frequency exponent *n*_f_ and film CPE impedance *Z*_CPEf_ when fitting the data by EEC model 1 for **PTA-1** (electronic supplementary material, table S1). It was found that *C*_dlf_ decreased from 5864.4 (for blank) to 107.9 (for 175 ppm **PTA-1** solution). This decrease is expected with an increase in film coating as the concentration of inhibitors increased from 0 to 175 ppm.

#### 3.2.1. Bode and Bode phase angle

**Figure 9.**
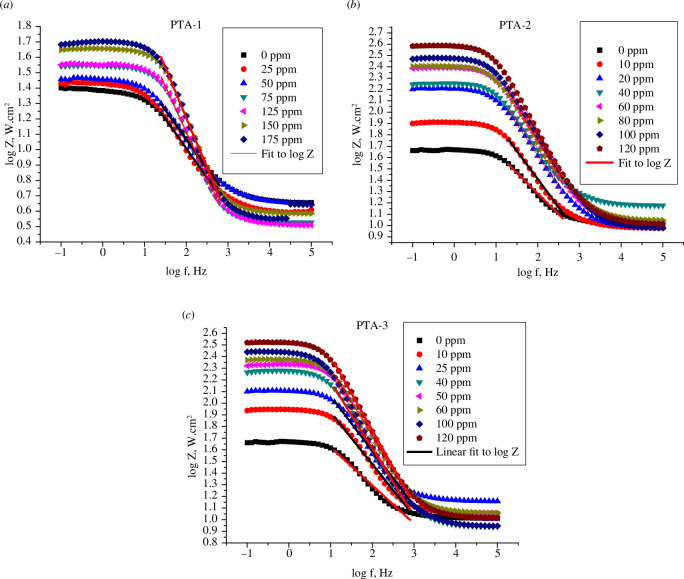
Bode plots of a C-steel electrode in 0.25 M H_2_SO_4_ solution with different concentrations of (*a*) **PTA-1**, (*b*) **PTA-2** and (*c*) **PTA-3**.

In a Bode plot, log |*Z*| was plotted against log *f*, where *Z* is the imaginary part of impedance in Ω cm^2^ and *f* is the frequency in Hz, for C-steel in 0.25 M H_2_SO_4_, in different concentrations of **PTA-1**, **PTA-2** and **PTA-3** (figure 9*a–c*) [69].

The values of surface irregularities for C-steel, *α*, were calculated from the slope of the linear region of the plot. Ideal capacitors have *α* value equal to −1 while coarse steel has *α* value less than −1. It is noted that the *α* values increased with concentration from −0.34 (0 ppm) to −0.65373 (175 ppm) for **PTA-1**; −0.6588 (120 ppm) for **PTA-2**; and −0.6341 (120 ppm) for **PTA-3** (table 4) [69].

The impedance modulus log|*Z*| at lowest frequency (0.1 Hz) showed an increasing trend as the concentration of triazine derivatives increased. In comparison with the blank sulfuric acid solution, the log|*Z*| value was improved by 1.18 orders of magnitude for the maximum concentration of 175 ppm of **PTA-1** (from 1.41 to 1.67), and even more than 1.56 orders of magnitude can be obtained for **PTA-2** (from 1.65 to 2.58) and 1.53 for **PTA-3** (from 1.65 to 2.52). This supported the formation of more rigid adsorption layer and thus higher inhibition efficiency of **PTA-2** and **PTA-3** than **PTA-1** for C-steel corrosion in sulfuric acid medium [73]

**Figure 10.**
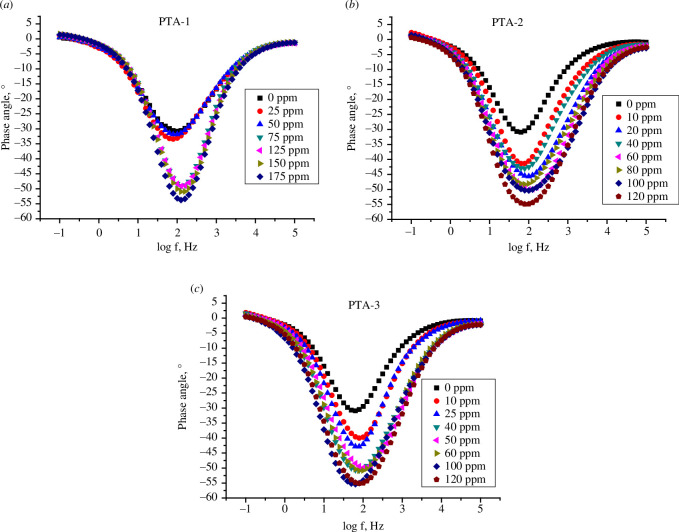
Bode phase angle plots of a C-steel electrode in 0.25 M H_2_SO_4_ solution with different concentrations of (*a*) **PTA-1**, (*b*) **PTA-2** and (*c*) **PTA-3**.

Figure 10*a–c* for **PTA-1**, **PTA-2** and **PTA-3** shows log Bode phase angle (°) plotted against log *f* (Hz) for the inhibitors. It is obvious that inhibition improved with increased inhibitor concentration since the phase angle values at the peak are more negative at higher concentrations. The phase angle values rose from −30.57° for the blank acid solution to −53.79° (**PTA-1** at 175 ppm), −54.89° (**PTA-2** at 120 ppm) and −55.12° (**PTA-3** at 120 ppm) (table 4) and thus the inhibitive behaviour increased [43,72].

### 3.3. Potentiodynamic polarization study

**Figure 11.**
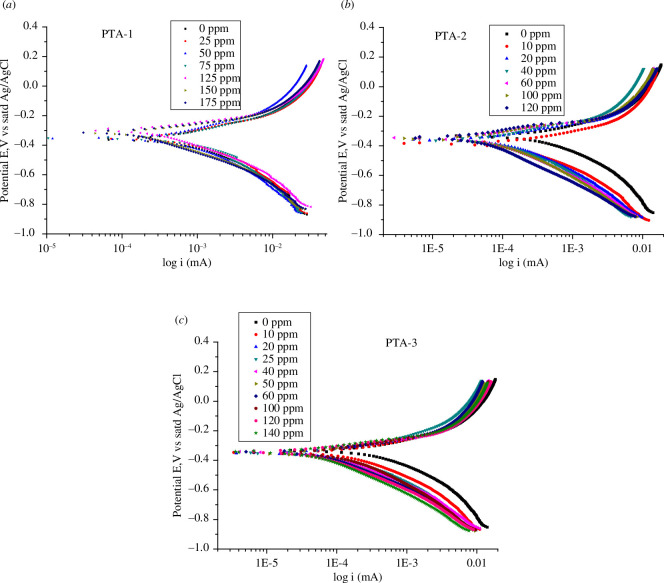
Semi-logarithmic polarization curves in 0.25 M H_2_SO_4_ at 298 K for all concentrations of (*a*) **PTA-1**, (*b*) **PTA-2** and (*c*) **PTA-3**.

The polarization curves of the C-steel in 0.25 M H_2_SO_4_ at various concentrations of inhibitors are shown in figure 11*a* for **PTA-1**, figure 11*b* for **PTA-2** and figure 11*c* for **PTA-3**.

The PDP electrochemical kinetics parameters were obtained from the polarization curve Tafel plot using Gamry software v. 7. The region chosen to draw the straight line was arbitrary. The variation of the anodic branch towards more positive potential with increasing concentration is not as obvious as in the case of cathodic branch towards more negative potential values. This consequently indicated that corrosion inhibition is mainly of cathodic type. The corrosion potential *E*_corr_ shifts in a random way to more negative values with an increase in inhibitor. The electrochemical corrosion kinetic parameters obtained from Tafel plots, corrosion potential (*E*_corr_), corrosion current density (*i*_corr_), corrosion rate (CR), cathodic and anodic Tafel slopes (*β*_c_ and *β*_a_), are listed in table 5 for **PTA-1**, table 6 for **PTA-2** and table 7 for **PTA-3** [74]. It seems that the *E*_corr_ values do not vary greatly with the inhibitor concentrations of **PTA-2**; the average value is −369.1 mV in the range 10–120 ppm (table 6). However, for **PTA-1**, *E*_corr_ decreased from −360.0 at 0 ppm to −293.0 at 75 ppm. Then increased slightly to −310.0 at 175 ppm (table 5). *E*_corr_ also decreased to −342.0 at 40 ppm of **PTA-3**, then changed very slightly at higher concentrations (table 7). In general, the corrosion potential *E*_corr_ shift in a random way to more negative values with increase inhibitor concentration could be related to enhancement in inhibition [69].

**Table 5.** Polarization parameters for various concentrations of for **PTA-1** on C-steel in 0.25 M H_2_SO_4_ medium (±error).

Tafel PTA-1	*β*_a_ (V/decade)	*β*_c_ (V/decade)	*i*_corr_ (μA)	*E*_corr_ ±s.d. (μV ± 21)	corrosion rate (mpy)	*Χ* ^2^	*i*_corr_ ±s.d. (μA cm^−2^ ± 51.4)	*θ*	IE% ± 0.65
0	0.3037	0.3822	1450	−360	1315	11.88	2884.4	0.000	0.000
25	0.2468	0.3275	1230	−352	1119	8.246	2446.8	0.152	15.2
50	0.3190	0.3774	1180	355	1076	10.76	2347.3	0.186	18.6
75	0.2293	0.3082	686	−293	623.8	32.42	1364.6	0.527	52.7
125	0.2117	0.2673	438	−297	397.7	53.7	871.3	0.698	69.8
150	0.2112	0.2716	385	−314	350.3	43.81	765.9	0.734	73.4
175	0.2017	0.2542	305	−310	277.2	48.6	606.7	0.79	79

**Table 6.** Polarization parameters for various concentrations of **PTA-2** on C-steel in 0.25 M H_2_SO_4_ medium (±error).

Tafel PTA-2	*β*_a_ (V/decade)	*β*_c_ (V/decade)	*i*_corr_ (μA)	*E*_corr_ ± s.d. (μV ± 25)	corrosion rate (mpy)	*Χ* ^2^	*i*_corr_ ± s.d. (μA cm^−2^ ± 25)	*θ*	IE% ± 0.60
0	0.3037	0.3822	1450	−360	1315.00	11.88	2884.4	0.000	0.0
10	0.2497	0.3232	316	−384	287.50	31.7	628.6	0.782	78.2
20	0.2294	0.3154	212	−360	192.40	23.93	421.7	0.854	85.4
40	0.2494	0.341	202	−360	183.50	21.05	401.8	0.861	86.1
60	0.2009	0.2813	113	−347	102.30	31.41	224.8	0.922	92.2
80	0.2056	0.2872	106	−398	96.80	31.79	210.9	0.927	92.7
100	0.1782	0.2652	70	−345	63.55	34.45	139.1	0.952	95.2
120	0.173	0.2474	51	−390	46.34	39.42	101.4	0.965	96.5

**Table 7.** Polarization parameters for various concentrations of **PTA-3** on C-steel in 0.25 M H_2_SO_4_ medium (±error).

Tafel PTA-3	*β*_a_ (V/decade)	*β*_c_ (V/decade)	*i*_corr_ (μA)	*E*_corr_ ± s.d. (μV ± 22)	corrosion rate (mpy)	*Χ* ^2^	*i*_corr_ ± s.d. (μA cm^−2^ ± 27)	*θ*	IE% ± 0.55
0	0.3037	0.3822	1450	−360	1315	11.88	2884.4	0	0
10	0.2562	0.3332	336	−346	305.3	11.88	668.4	0.768	76.8
25	0.2488	0.3185	225	−350	204.4	20.17	447.6	0.845	84.5
40	0.2089	0.2701	139	−342	126.6	37.72	276.5	0.904	90.4
50	0.2161	0.284	140	−352	126.9	31.76	278.5	0.903	90.3
60	0.2172	0.2838	133	−345	120.6	34.56	264.6	0.908	90.8
100	0.2004	0.2626	115	−339	104.9	41.2	228.8	0.921	92.1
120	0.1908	0.2601	92	−342	83.72	37.49	183.2	0.936	93.6

On the other hand, the decrease in current density *i*_corr_ (μA cm^−2^) and corrosion rates CR (mpy) was obvious for all three compounds, where mpy is mils per year. *i*_corr_ and CR decrease from 1450.0 and 1315.00 for blank solution to 305.00 and 277.20 for **PTA-1** at 175 ppm; 51.0 and 46.34 for **PTA-2** at 120 ppm; 92.1 and 83.72 for **PTA-3** at 120 ppm. Thus, **PTA-2** showed the highest inhibition efficiency among the three compounds studied since it has the lowest *i*_corr_ and CR at 120 ppm. This indicates that the *para*-bromo group on the phenyl of triazine compound **PTA-2** affects *i*_corr_ and CR strongly, then followed by the *para*-methoxy group in the case of **PTA-2**. Thus, the inhibition efficiency IE% of anticorrosion follows the order **PTA-2** (96.5%) > **PTA-3** (93.4%) > **PTA-1** (79.0%).

Increasing the amounts of inhibitors shifted the cathodic curves more than the anodic curves to smaller corrosion current density (*i*_corr_) (figure 11*a*–*c*). Thus, the addition of the inhibitors affected the cathodic proton-discharge (hydrogen-evolution) reaction more than the rate of anodic iron dissolution. This indicated that anticorrosion inhibition is a mixed type dominated by cathodic inhibition [40].

It can be seen from tables 5–7 that the values of cathodic slope *β*_c_ (mV dec^−1^) and anodic slope *β*_a_ (mV dec^−1^) decreased with increasing inhibitor concentrations. The decrease extent of *β_c_* and *β*_a_ was in the order **PTA-2** > **PTA-3** > **PTA-1**. The *β*_c_ values at the highest concentration of inhibitors were 0.2542, 0.2474 and 0.2601 (mV dec^−1^) for **PTA-1**, **PTA-2** and **PTA-3**, respectively. This indicated that **PTA-2** showed better inhibitory efficiency compared to the other two inhibitors [64]. The inhibition efficiency IE% and the surface coverage *θ* values were calculated from corrosion current density (*i*_corr_) values as described in [69,73]. The inhibitory efficiency IE% increased with the increase in inhibitor concentration, reaching the greatest values of 79.0% for **PTA-1** at 175 ppm, 96.5% for **PTA-2** at 120 ppm and 93.6% for **PTA-3** at 120 ppm (tables 5–7). These values are in good agreement with the results from impedance studies except for **PTA-1**.

### 3.4. Adsorption isotherm

**Figure 12.**
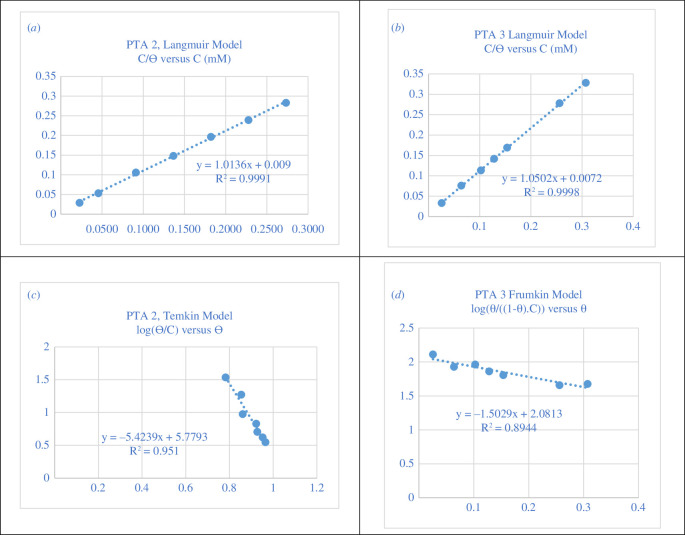
Langmuir adsorption isotherm of (*a*) **PTA-2** and (*b*) **PTA-3**; (*c*) Temkin adsorption isotherm of **PTA-2**; (*d*) Frumkin adsorption isotherm of **PTA-3** for all concentrations on carbon steel electrode in 0.25 M H_2_SO_4_ at 298 K.

The experimental data were fitted to Langmuir, Temkin and Frumkin isotherms. The Langmuir adsorption isotherm equation (3.1) related to the surface coverage (*θ*) and the concentration of inhibitor (*C*) as [42,43]:


(3.1) C/θ=1/K+C,

where *C* (mM) is the inhibitor concentration and *K*_ads_ is the adsorption equilibrium constant. The straight lines obtained by plotting *C*/*θ* versus *C* in mM [62] are shown in figure 12*a,b* . The data fitted well with the Langmuir adsorption isotherm for **PTA-2** and **PTA-3** with regression coefficient *R* ^2^ = 0.9991 and 0.9998, respectively. While Langmuir fitting for **PTA-1** gave *R* ^2^ of only 0.7305. The slope values for **PTA-2** and **PTA-3** are 1.014 and 1.050, close to 1.00 the theoretical value expected from the Langmuir equation.

The fitting of the Temkin model was carried by plotting log(*θ*/*C*) versus *θ*, according to equation (3.2) (figure 12*c*) [74]:


(3.2) log⁡(θ/C)=log⁡K−gθ,

where *g* is the adsorbate parameter. The *R^2^* values obtained from the straight line for **PTA-1**, **PTA-2** and **PTA-3** are 0.3933, 0.9510 and 0.8852, respectively. Thus, the Temkin model is less acceptable than Langmuir model.

The data were also fitted to the Frumkin model (figure 12*d* and electronic supplementary material, figure S4), equation (3.3) [74]:


(3.3) $$\log[\theta /(1-\theta )C]=\log K_{ads}+g\theta .$$

The plot of log[*θ*/(1 − *θ*)*C*)] versus *θ* gave *R* ^2^ values of 0.3503 and 0.8944, also less than that of the Langmuir model, for **PTA-2** and **PTA-3** (figure 12*d*). But, Frumkin model *R* ^2^ is 0.8678 for **PTA-1** which is higher than that obtained by the Langmuir model (electronic supplementary material, figure S4). Thus, the Langmuir model is suitable for **PTA-2** and **PTA-3** while the Frumkin model is more suitable for **PTA-1** (electronic supplementary material, figure S2).

The standard free energy of adsorption (Δ*G°*_ads_) is related to the adsorption constant (*K*_ads_) by the following equation [42,43,75]:


(3.4) $$\Delta G_{ads}^{\circ }=-RT\ln(55.5K_{ads}),$$

where *R* is the universal gas constant, *T* is the absolute temperature and 55.5 is the molar concentration of water in the solution. *K*_ads_ values were found equal to 2.73 × 10^3^, 1.11 × 10^5^ and 1.39 × 10^5^ for **PTA-1**, **PTA-2** and **PTA-3**, respectively. The calculated values of Δ*G°*_ads_ on C-steel are −38.76 kJ mol^−1^ for **PTA-2** and −39.32 kJ mol^−1^ for **PTA-3**. In the case of **PTA-1**, Δ*G*°_ads_ was found to be −29.58 kJ mol^−1^ [43]. This indicated that adsorption of inhibitor on steel surface is favoured over its presence in solution [76].

The *para* substituents –Br and –OCH_3_ on the aniline moiety of **PTA-2** and **PTA-3**, respectively, are polar which has the effect of increasing the solubility of inhibitors in aqueous acidic media. Also, the presence of lone electron pairs on the *para* substituents rendered the inhibitors to act as Lewis bases that can donate electrons to Fe of the electrode surface. This is supported by very high negative Δ*G°*_ads_ value of **PTA-2** and **PTA-3**. While **PTA-1** with no *para* substituent had less interaction with Fe surface and showed smaller Δ*G°*_ads_ values.

### 3.5. Adsorption mechanism

**Figure 13.**
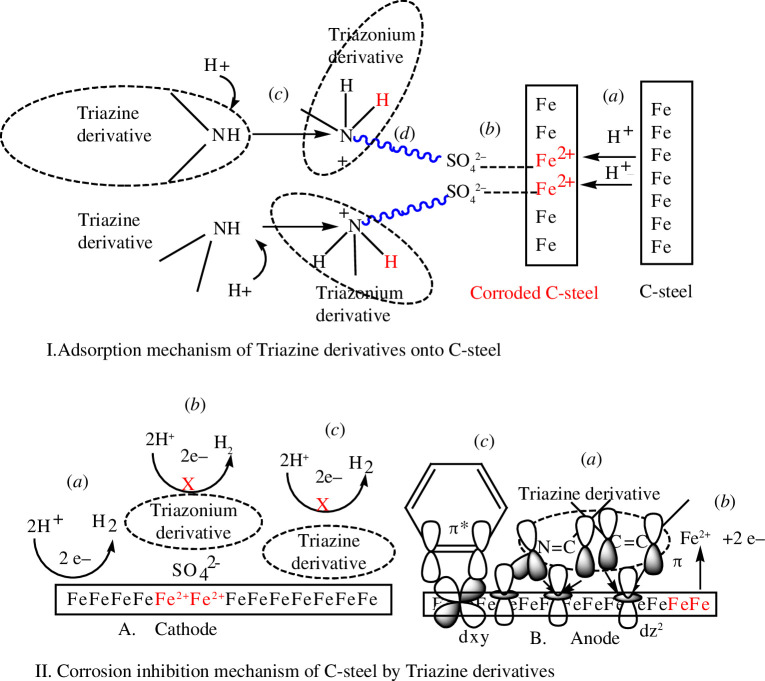
Proposed representation of (I) physical adsorption of triazines onto C-steel in 0.25 M H_2_SO_4_ solution and (II) inhibition mechanism at (A) the cathode and (B) the anode as well as physical adsorption (A. ***b***) and chemical adsorption (B. ***a***) and (*c*).

Physical adsorption of the triazine derivatives is caused by electrostatic interactions between the protonated compounds **PTA-1**, **PTA-2** and **PTA-3**, and the negatively charged steel surface as shown in figure 13I [43,72].

— First, iron cation Fe^2+^ is formed by oxidation of Fe as a result of attack of acid H_2_SO_4_ onto the surface of C-steel.
— SO_4_^2−^ anion from H_2_SO_4_ is attracted to the positively charged steel surface containing Fe^2+^ through electrostatic interactions, causing the surface of C-steel to become negatively charged
— The triazine derivatives **PTA-1**, **PTA-2** and **PTA-3** are converted to positively charged triazonium forms by the attack of proton H^+^.
— The positive triazonium species adsorb onto negatively charged C-steel via electrostatic interaction.

Chemical adsorption of the triazine derivatives onto the C-steel surface occurred through formation of coordinate bonds between electron-rich functional groups of triazine with empty *d*-orbital of Fe atom or Fe^2+^ ion. >C=N of imine of triazine and diazole rings, and >C=C< of phenyl ring group can share π electrons. The presence of methyl groups (electron donor by inductive effect) on the imidazole ring in place of H atoms increases electron donation of functional groups. While –N= and N< can share free electron pair as in **PTA-1** (figure 13IIB*a)*. **PTA-2** and **PTA-3** have additionally extra free electron pair donor groups, –Br and –OMe, respectively [44].

Chemical adsorption of the triazine onto C-steel surface can also occur through π-electron back donation from Fe *d*_t2g_ orbital onto empty π* orbital of aromatic rings of *s*-triazines: phenyl amine derivative, triazine and diazole (figure 13IIB*c*). But, Br is stronger coordinating with Fe^2+^ or Fe than OMe. This can explain the little increase in the inhibition efficiency of **PTA-2** compared to **PTA-3**.

### 3.6. Inhibition mechanism

Corrosion inhibition of C-steel in sulfuric acid solution by different triazines can be due to adsorption. The triazines inhibit corrosion by blocking both anodic and cathodic sites. In weak acidic solution, the triazines exist as neutral or protonated species.

The neutral triazines are chemically adsorbed on steel while protonated triazines are physically adsorbed on the cathodic sites of the mild steel forming a protective film (figure 13IIA *b*,*c*). This leads to a decrease in the exposed surface area of C-steel with the acid solution and a decrease in the evolution of hydrogen, as shown in figure 13IIA [41].

The adsorption of triazines on anodic site occurs through π electrons of imines and aromatic rings and through lone pair of electrons of various heteronitrogen atoms, and additionally of bromine in **PTA-2** and oxygen (from methoxy group) in **PTA-3**. This decreases the anodic dissolution of C-steel [76].

In conclusion, the high performance of *s*-triazines is attributed to the presence of π electrons, lone pair electrons from nitrogen atoms of *s*-triazine and diazole rings, and quaternary nitrogen atoms, nd large molecular size and the planarity of these compounds. Both planarity and larger molecular size of studied molecules ensure formation of larger protective film on mild steel. Also, electron donation by inductive effect from methyl groups and by resonance from bromine and methoxy groups increases electron density at triazines causing stronger adsorption/inhibition on the metal surface [42–44]. The order of inhibition efficiency is: **PTA-2** ≈ **PTA-3** > **PTA-1**.

### 3.7. Comparative studies of inhibition by different symmetrical *s*-triazines

The anticorrosion application of *s*-triazine derivatives has been studied recently. We present, in table 8, the experimental results of inhibition by selected triazine: IE% inhibition from Tafel plot, maximum dose used, techniques used, molecular structure, adsorption type and isotherm for steel in 1 M HCl [40,42–44,72,77]. It is obvious from table 8 that the structural effect of triazine molecules, such as the functional groups, alkyl side chains, presence of heteroatoms, phenyl rings, π-bond conjugation, affects the overall corrosion inhibition efficiency.

**Table 8.** *s*-Triazine-based corrosion inhibitors: structure, IE% from Tafel plot, maximum dose, adsorption type and isotherm for steel in 1 M HCl; while PTA-1, -2 and -3 in 0.25 M H_2_SO_4_.

**s-triazine derivatives structures** 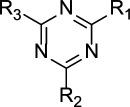	**metal/medium**	**concentration IE%**	**adsorption isotherm (type)**	**reference**
R_1_=R_2_=R_3_ = 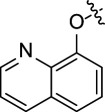 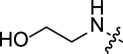 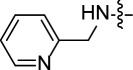	steel/1 M HCl	(250 ppm)	Langmuir (mixed)	[43]
		98.8%		
		85.1%		
		96.9%		
R_1_=R_2_=R_3_ = 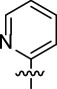	mild steel/1 M HCl	(312 ppm) 89.3%	Langmuir (mixed)	[40]
R_1_ =R_3_ = NH_2_NH–; R_2_ = 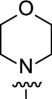 R_2_ = 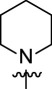	steel/1 M HCl	(225 ppm)	Langmuir (mixed)	[42]
		98%		
		97%		
R_1_ = R_3_ = NH_2_–NH– ; R_2_ = CH_3_O– R_1_ = NH_2_–NH– ; R_2_ = R_3_ = CH_3_O– R_1_ = R_2_ = R_3_ = NH_2_–NH–	steel/1 M HCl	(250 ppm)	Langmuir (mixed)	[44]
		98.0%		
		95.9%		
		97.7%		
R_1_=R_2_=R_3_ = 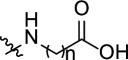 *n* = 1 *n* = 2 *n* = 3 *n* = 4 *n* = 5	mild steel/1 M HCl	(5000 ppm) 71.8%	Langmuir (mixed)	[77]
		(5000 ppm) 76.5%		
		(5000 ppm) 81.7%		
		(2000 ppm) 92.0%		
		(500 ppm) 98.4%		
R_1_=R_2_=R_3_ = 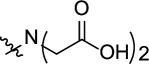	mild steel/1 M HCl	(5000 ppm) 35.4%	Langmuir (mixed)	[72]
R_1_=R_3_= 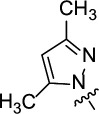 R_2_ = 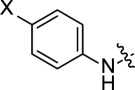 X = H X = Br X = OCH_3_	C-steel/0.25 M H_2_SO_4_	(175 ppm) 79.0%	Frumkin (mixed)	present work
		(120 ppm) 96.5%	Langmuir (mixed)	
		(120 ppm) 93.4%	Langmuir (mixed)	

### 3.8. Density functional theory analysis

#### 3.8.1. Geometry optimization

**Figure 14.**
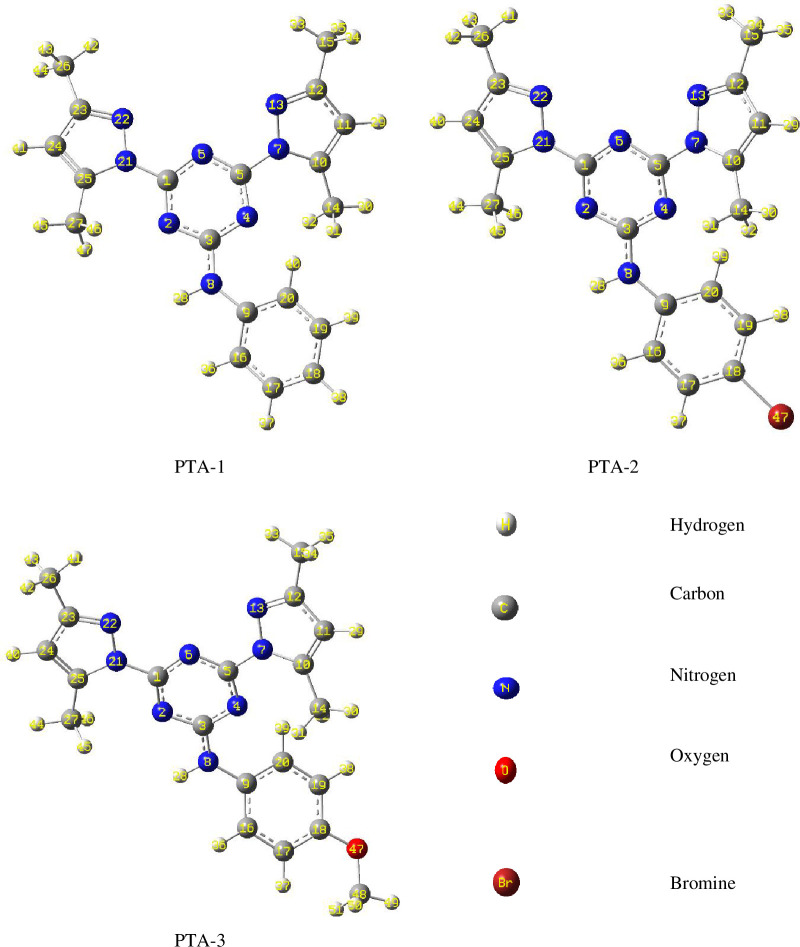
Optimized structures indicating numbering scheme for the atoms of the stable conformers, for the neutral form of synthesized organic heterocycles **PTA-1**, **PTA-2** and **PTA-3** (calculated at the B3LYP/6-311G++(**d,p**) level).

The optimized structures of the synthesized compounds are presented in figure 14. The molecule **PTA-3** can possibly exist in two different isomers due to the presence of –OCH_3_ substituent, so both isomers were examined by DFT calculations to determine the most stable conformer. The studied compounds are structurally similar; however, presence of –Br and –OCH_3_ substituents on C-18 of **PTA-2** and **PTA-3**, respectively, provide additional features for the comparative investigation.

#### 3.8.2. Frontier molecular orbitals and electrostatic potential map analysis

**Figure 15.**
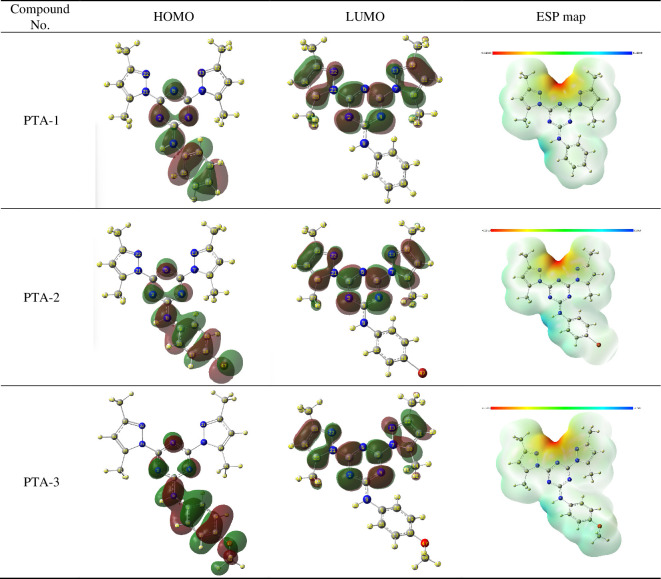
Frontier molecular orbitals (HOMO and LUMO) and ESP maps of the stable conformers for the neutral form of compounds **PTA-1, PTA-2 and PTA-3** by B3LYP/6-311G++(**d,p**) calculations. FMOs and ESP maps for the protonated form of compounds **PTA-1, PTA-2 and PTA-3** by B3LYP/6-311G++(**d,p**) calculations are also provided in electronic supplementary material, figure S5.

In order to gain a better insight into the chemical behaviour of organic molecules such as reactivity and stability, it is essential to analyse their frontier molecular orbitals (FMOs). These comprise HOMO and LUMO. The energy difference between these molecular orbitals is termed as energy gap or band gap, which is quite sensitive to the molecular substituents. The energy of HOMO (*E*_HOMO_) refers to the molecular tendency to donate electrons. Hence, high values of *E*_HOMO_ reflect the molecule’s tendency to donate electrons to electrophilic moieties, in addition to its direct correlation with the ionization potential of a molecule. On the other hand, the low energy of LUMO (*E*_LUMO_) indicates the ability to gain electrons which enables one to detect electrophilic sites in a molecule and that is related to its electron affinity. As illustrated in figure 15, the frontier HOMO and LUMO orbital surfaces for the three molecules reported in this study are alike. For all the compounds, it is clearly evident that the electron density on HOMO is primarily localized on the phenylamine motif and extended to N atoms of the triazine structure. In the case of LUMO, the electron density is located on the pyrazole and triazine moieties of the molecules. Molecular electrostatic potential (ESP) maps of the three molecules are also highlighted in figure 15, which displays a three-dimensional distribution of the molecular electronic cloud. Electron-rich sites of the molecule are coloured in red which refers to the region with lowest ESP, and that appears on the triazine ring having the highest electronic density in all three compounds. While the electron-deficient sites show the highest ESP and are coloured in blue.

#### 3.8.3. *E*_HOMO_, *E*_LUMO_ and Δ*E*

The energy values of FMOs were calculated for both neutral and protonated states of all three compounds in both gaseous and aqueous phases, which are presented in table 9. According to the observed values in both neutral and protonated states, the largest *E*_HOMO_ and the lowest *E*_LUMO_ values were obtained for the substituted compounds, **PTA-3** and **PTA-2**, respectively. As a result, the energy gap (Δ*E*) followed a descending pattern from **PTA-1** > **PTA-2** > **PTA-3** for both neutral and protonated forms of the compounds. In general, low value of Δ*E* and high value of *E*_HOMO_ are linked with high corrosion inhibition efficiency. A low energy difference value results in a facile electron removal from the last occupied orbital for donation to the unoccupied d orbitals of the metal atom. Higher value of *E*_HOMO_ is linked with the greater ability of a molecule to donate an electron to the metal surface which maximizes the interaction with the metal. This leads to a better adsorption and therefore enhances the corrosion inhibition efficiency. Based on the results tabulated in table 9, it is expected that there will be a better corrosion inhibition efficiency for both **PTA-3** and **PTA-2** compared with **PTA-1** which is further confirmed by the experimental results.

**Table 9.** *E*_HOMO_, *E*_LUMO_ and Δ*E* (in eV) for the neutral and protonated forms of **PTA-1**, **PTA-2** and **PTA-3** calculated in the gaseous and aqueous (aq.) phases at the B3LYP/6-311G++(d,p) level of theory.

compound	phase	neutral form			protonated form		
		*E*_HOMO_	*E*_LUMO_	Δ*E*	*E*_HOMO_	*E*_LUMO_	Δ*E*
**PTA-1**	gas	−6.373	−1.604	4.769	−9.685	−5.292	4.393
	aq.	−6.443	−1.78	4.663	−7.058	−2.559	4.499
**PTA-2**	gas	−6.401	−1.713	4.688	−9.3	−5.37	3.93
	aq.	−6.393	−1.822	4.571	−6.911	−2.616	4.295
**PTA-3**	gas	−6.015	−1.49	4.525	−8.805	−5.181	3.625
	aq.	−6.077	−1.731	4.347	−6.469	−2.502	3.967

#### 3.8.4. Quantum chemical parameters

The reactivity and stability of molecules are indicated by the global reactivity descriptors which can be computed using ionization potentials (*I*) and electron affinities (*A*). Ionization potential refers to the amount of energy needed to remove one electron from a molecule in the gaseous phase, while the addition of an electron to a neutral molecule is known as the electron affinity which is computed in the form of a released energy. Despite the generally adopted Koopmans’ theorem [78] which equates the ionization potential with the energy of HOMO and electron affinity with the LUMO energy, a more accurate approach is to consider vertical ionization potentials (*I*) and electron affinities (*A*) based on obtaining the excitation energetics, and these are calculated using equations (3.5) and (3.6), respectively [79]:


(3.5) I=[E(radicalcation)−E(neutralmolecule)].

(3.6) A=[E(neutralmolecule)−E(radicalanion)].

High value of ionization energy reflects chemical inertness and high stability while small ionization energy reflects high reactivity of the atoms and molecules. The values of *I* and *A* can be utilized to compute other chemical descriptors that rely on these quantities including electronegativity, electron chemical potential, global hardness and softness, electrophilicity index, electron back donation and fraction of electron transfer. Electronegativity (*χ*) measures the tendency of an atom to attract electrons and is described by the arithmetic mean of *I* and *A*. Accordingly, electrons tend to flow from molecules with low electronegativity towards species with high electronegativity and molecules with low χ are generally linked with greater inhibition efficiency. It is measured using the formula of equation (3.7):


(3.7) $$\chi =\frac{I+A}{2}.$$

Electronic chemical potential (*µ*) measures the escaping tendency of electron from equilibrium and a compound with high chemical potential is considered as a good electron donor, whereas a molecule having small chemical potential is generally a good electron acceptor. It is defined as the inverse of the electronegativity (*χ*).

Both global hardness (*η*) and global softness (*S*) are important quantum chemical descriptors which provide useful information about stability and reactivity of a molecule. The softness of a molecule refers to its high reactivity that highlights a facile electron transfer to an electron acceptor. It is the inverse of the hardness and reflects the presence of a small energy gap between the FMOs compared to the hard species. Therefore, hard substances are considered more stable and with less of a tendency to undergo any chemical changes. In addition, the chemical reactivity is directly proportional to the softness value and linked inversely with the hardness value. These parameters are calculated using equations (3.8) and (3.9):


(3.8) $$\eta =\frac{I-A}{2}.$$

(3.9) $$S=\frac{1}{\eta }.$$

The electrophilicity (ω*) indicates the ability of the inhibitor molecules to accept electrons. It reflects stabilization in energy after a molecule accepts an additional amount of electron charge from the environment. Higher value of electrophilicity means that the compound would be stabilized by a nucleophilic attack and vice versa. Also, high value of electrophilicity is a characteristic of a good electrophile while lower value of electrophilicity indicates a good and more reactive nucleophile. It is measured using equation (3.10) which is based on electronegativity and hardness:


(3.10) $$\omega^{*}=\frac{\chi^{2}}{2\eta }.$$

Equations (3.11) and (3.12) are used to calculate the electron donor capacity (*ω* ^–^) and electron acceptor capacity (*ω* ^+^) which measure the tendency of a molecule to donate and accept charge, respectively.


(3.11) $$\omega^{-}=\frac{{\left(3I+A\right)}^{2}}{16\left(I-A\right)}.$$

(3.12) $$\omega^{+}=\frac{{\left(I+3A\right)}^{2}}{16\left(I-A\right)}.$$

Electron back donation (*E*_B-d_) is defined as the study of the interaction between the metallic surface and the corrosion inhibitor through charge transfer process. The energy change (Δ*E*_B-d_) of this process is directly proportional to the stability/hardness of the molecule which is measured as follows:


(3.13) $$\Delta E_{B\text{-}d}=-\frac{\eta }{4}.$$

Low *E*_B-d_ values indicate high molecular stability, which can lead to reduce the inhibition potency. Dipole moment (*D*) is also an important parameter that provides information regarding bond polarity and the distribution of the electronic cloud within a molecule. Large values of *D* signify unbalanced electronic distribution leading to substantial adsorption of the organic inhibitor on the metallic surface and, thus, enhance inhibition efficiency.

The number of transferred electrons (Δ*N*) from a species with lower electronegativity (inhibitor molecule) to another species with higher electronegativity (iron surface) is estimated using equation (3.14):


(3.14) $$\Delta N=\frac{\left(\chi_{Fe}-\chi_{mol}\right)}{2\left(\eta_{Fe}+\eta_{mol}\right)}.$$

In equation (3.14), *χ*_Fe_ and *η*_Fe_ represent the theoretical electronegativity and hardness values of the iron surface, respectively, while *χ*_mol_ and *η*_mol_ denote the calculated values of electronegativity and hardness for the molecule being studied as corrosion inhibitor. The theoretical values for the iron surface are *χ*_Fe_ = 7.0 eV mol^−1^ and *η*_Fe_ = 0 eV mol^−1^ by assuming that for a metallic bulk *I* = *A* as it is softer than the neutral metallic atoms [80].

The quantum chemical descriptors calculated using equations (3.5)–(3.14) for **PTA-1**, **PTA-2** and **PTA-3** in gaseous and aqueous phases are tabulated in table 10. The calculated ionization potential (*I*) values in the aqueous phase are in the order of **PTA-1** (6.32 eV) > **PTA-2** (6.27 eV) > **PTA-3** (5.98 eV). A similar trend is observed for the calculations carried out in the gaseous phase. As lower value of *I* is associated with high reactivity, **PTA-3** is expected to exhibit higher corrosion inhibition efficiency compared to the other two inhibitor molecules. Similar trend is observed for electronegativity (*χ*), global hardness (*η*) and electrophilicity (ω*) where the values are following a descending order as **PTA-1** > **PTA-2** > **PTA-3**. Like the small value of *I*, small values of *χ*, *η* and ω* are also associated with higher inhibition efficiency which suggests the highest inhibition efficiency for **PTA-3**. As expected, the value of softness (*S*) is: **PTA-3** > **PTA-2** > **PTA-1**, as it is inverse of hardness. The highest values for the dipole moment (*D*) and electronic back donation were also found for the inhibitor **PTA-3**. One of the most important factors to measure the corrosion inhibition efficiency is the fraction of electron transfer (Δ*N*) and obtained values for this also agree with Lukovit’s study [80]. According to this, if Δ*N* < 3.6, the corrosion inhibition efficiency increases by increasing the electron-donating ability of corrosion inhibitors at the mild steel or electrolyte interface. All the calculated values of Δ*N* are below 3.6 and **PTA-3** has the highest value of 0.44 and 0.75 in gaseous and aqueous phases, respectively, while **PTA-1** has the lowest values of Δ*N*, 0.40 and 0.66, corresponding to calculations performed in gaseous and aqueous phases, respectively. This correlates strongly with the experimental inhibition efficiency results where **PTA-1** was the least efficient corrosion inhibitor among the studied compounds while both **PTA-2** and **PTA-3** have shown better corrosion inhibition efficiency. A similar trend showing better corrosion inhibition efficiency by **PTA-3** compared to **PTA-2** and **PTA-1** was observed when the calculations were performed for the protonated forms of these three compounds at the same level of theory.

**Table 10.** The calculated chemical parameters for the neutral form of **PTA-1**, **PTA-2** and **PTA-3** calculated at the B3LYP/6-311G++(d,p) level in gaseous and aqueous (aq.) phases and protonated form of **PTA-1**, **PTA-2** and **PTA-3** in aqueous (aq.) phase by employing excitation energetics.

chemical parameters	PTA-1			PTA-2			PTA-3		
	gas	aq.	protonated* (aq.)	gas	aq.	protonated* (aq.)	gas	aq.	protonated* (aq.)
electronic energy (neutral, hartrees)	−1174.389	−1174.409	−1174.856	−3747.93	−3747.952	−3748.398	−1288.944	−1288.967	−1289.415
electronic energy (radical cation, hartrees)	−1174.105	−1174.177	−1174.601	−3747.647	−3747.721	−3748.147	−1288.671	−1288.747	−1289.180
electronic energy (radical anion, hartrees)	−1174.399	−1174.48	−1174.956	−3747.946	−3748.024	−3748.500	−1288.951	−1289.036	−1289.512
Ionization potential (*I*, eV)	7.72	6.32	6.95	7.71	6.27	6.81	7.42	5.98	6.38
electron affinity (*A*, eV)	0.27	1.94	2.71	0.42	1.98	2.77	0.2	1.89	2.65
electronegativity (*χ*, eV)	3.99	4.13	4.83	4.07	4.12	4.79	3.81	3.93	4.52
electronic chemical potential (*µ*, eV)	−3.99	−4.13	−4.83	−4.07	−4.12	−4.79	−3.81	−3.93	−4.52
global hardness (*η*, eV)	3.72	2.19	2.12	3.65	2.14	2.02	3.61	2.05	1.87
global softness (*S*, eV)	0.27	0.46	0.47	0.27	0.47	0.5	0.28	0.49	0.54
electrophilicity (ω*, eV)	29.69	18.68	24.67	30.15	18.24	23.12	26.24	15.85	19.04
electron donor capacity (*ω* ^–^, eV)	4.61	6.22	8.18	4.75	6.3	8.33	4.37	6	7.96
electron acceptor capacity (*ω* ^+^, eV)	0.61	2.1	3.36	0.69	2.17	3.54	0.56	2.07	3.44
electronic back donation (Δ*E*_B-d_, eV)	−0.93	−0.55	−0.53	−0.91	−0.54	−0.5	−0.9	−0.51	−0.47
dipole moment (*D*)	3.961	6.615	1.615	2.892	5.242	8.918	5.786	8.921	3.364
fraction of electron transfer (Δ*N*)	0.4	0.66	0.51	0.4	0.67	0.55	0.44	0.75	0.67

At this juncture, quantum chemical parameters for all three compounds were calculated using widely reported Koopmans’ theorem [78] and compared with the values obtained by employing a more accurate method based on vertical ionization potentials (*I*) and electron affinities (*A*) as reported in table 10. According to Koopmans’ theorem, *E*_HOMO_ and *E*_LUMO_ are directly associated with ionization potentials (*I*) and electron affinities (*A*) according to equations (3.15) and (3.16)


(3.15) $$I=-E_{HOMO}.$$

(3.16) $$A=-E_{LUMO}.$$

The data obtained for the quantum chemical parameters by employing Koopmans’ theorem are presented in electronic supplementary material, figure S6. As expected, the values for the studied descriptors are different for both approaches; however, the obtained trends by both methods are in good agreement with each other. Overall, in both neutral and protonated forms, compound **PTA-1** is the least efficient corrosion inhibitor as supported by the experimental results, while compound **PTA-3** showed the highest tendency to be an efficient corrosion inhibitor.

#### 3.8.5. Local selectivity by condensed Fukui function

Condensed Fukui function *f* is a well-recognized means to analyse local selectivity of an inhibitor molecule [81]. The values indicate the atoms in a molecule that are more susceptible to electrophilic or nucleophilic attacks. The preferred site for an attack by an electrophilic or nucleophilic agent will be at the atom where the value of $f_{k}^{-}$ or $f_{k}^{+}$ is maximum. The condensed Fukui functions are calculated using equations (3.17) and (3.18)


(3.17) $$f_{k}^{+}=q_{k}\left(N+1\right)-q_{k}\left(N\right)\;(nucleophilic\;attack),$$

(3.18) $$f_{k}^{-}=q_{k}\left(N\right)-q_{k}\left(N-1\right)\;(electrophilic\;attack),$$

where $q_{k}$ is the charge at atomic centre *k* and $q_{k}\left(N+1\right)$ , $q_{k}\left(N\right)$ and $q_{k}\left(N-1\right)$ are the electronic population of the atom *k* in the anionic, neutral and cationic species, respectively.

The difference between the nucleophilic and electrophilic Fukui functions is known as a dual descriptor $\left(∆f\right)$ [82]. It is given as follows:


(3.19) $$\Delta f=f_{k}^{+}-f_{k}^{-}.$$

The local softness (*s*) of an atom can be described as the product of the condensed Fukui function ( f ) and the global softness (*S*), where high values of $s^{+}$ indicate high nucleophilicity, while high values of $s^{-}$ are indication of high electrophilicity.


(3.20) $$s^{+}=(f^{+})S\;(nucleophilic\;attack),$$

(3.21) $$s^{-}=(f^{-})S\;(electrophilic\;attack).$$

Calculated values of Mulliken charges, condensed Fukui functions, dual descriptors and local softness indices for **PTA-1**, **PTA-2** and **PTA-3** are presented in electronic supplementary material, tables S5–S7 and figure S6, respectively. The numbering sequence for the atoms is highlighted in figure 14. It can be deduced that both N(7) and N(21) have the highest value for $f_{k}^{+}$ which means these are the most preferential reactive site for nucleophilic attack for all the compounds. For **PTA-3**, the $f_{k}^{+}$ values for N(7) and N(21) are equal, and for the compounds, the $f_{k}^{+}$ values for N(21) are similar to that of N(7) as well. This is expected as N(7) and N(21) are in the same environment. The most reactive electrophilic sites based on the obtained values of $f_{k}^{-}$ are C(14), C(3) and C(48) for **PTA-1**, **PTA-2** and **PTA-3**, respectively. In addition, the energies and the Cartesian coordinates of the optimized structures are provided in electronic supplementary material, tables S8 and S9. Further computational investigation is underway to explore other pertinent features of the corrosion inhibitors and will be documented in due course.

### 3.9. Monte Carlo simulation

**Figure 16.**
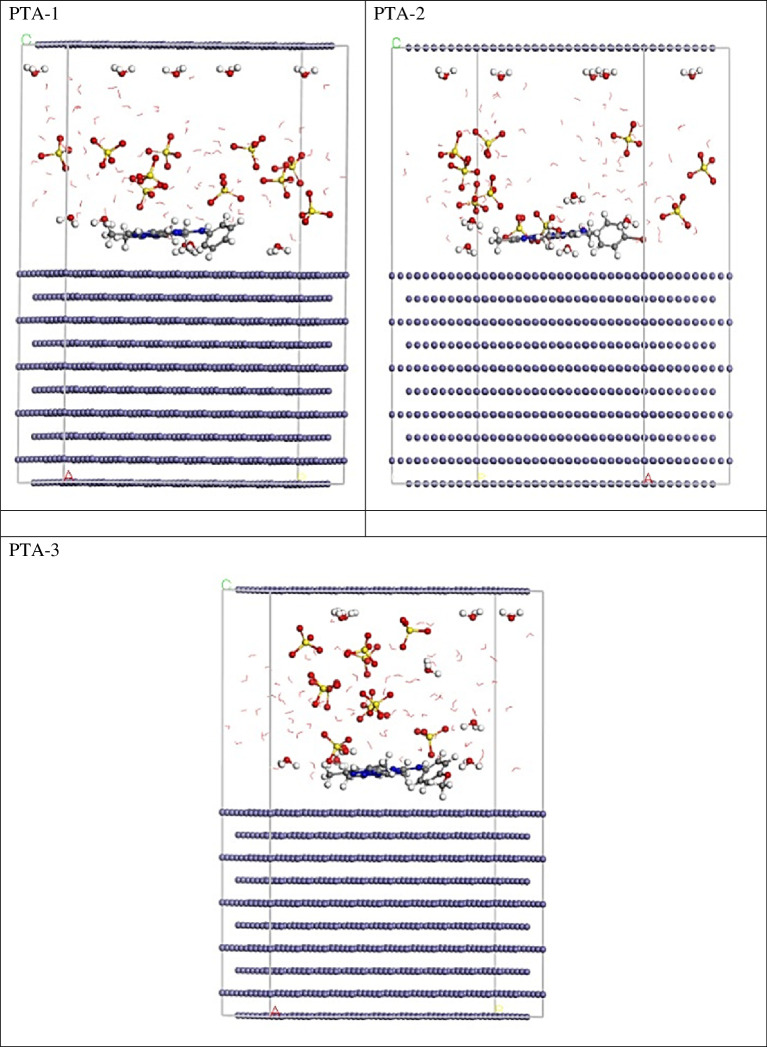
The highest proper adsorption arrangement for the **PTA-1**, **PTA-2** and **PTA-3** molecules on Fe(110) substrate achieved by adsorption locator module.

MC simulations were employed to investigate the interactions between the inhibitor molecules and the steel surface, aiming to elucidate the mechanism of the adsorption process. These simulations were specifically designed to analyse and understand the binding behaviour and overall interaction patterns between the inhibitor molecules and the steel surface. By using MC simulations, a clear understanding of the adsorption mechanism was obtained, providing valuable insights into how the inhibitor molecules interact with and bind to the steel surface. Fe(110) plane was used as a model for carbon steel surface in this work for MC simulations, since it is believed to be more stable energetically than the Fe(100) and Fe(111) surfaces [83]. Figure 16 illustrates the most favourable adsorption configurations for **PTA-1**, **PTA-2** and **PTA-3** molecules on the steel surface in a corrosive medium, as revealed by the adsorption locator module. The adsorption formations depicted in the figure exhibit a nearly flat arrangement, indicating an enhancement in adsorption efficiency and maximum surface coverage. This finding suggests that these adsorption configurations are highly suitable for achieving effective protection against corrosion on the steel surface [84]. Moreover, the calculated adsorption energies obtained from the MC simulations are documented in table 11. It was observed that **PTA-2** and **PTA-3** molecules exhibited higher negative adsorption energy values (−2112.34 and −2126.36 kcal mol^−1^, respectively) compared to the adsorption energy of **PTA-1** molecule (−1765.78 kcal mol^−1^). This suggests that **PTA-2** and **PTA-3** molecules undergo energetically favourable adsorption on the steel interface, forming a stable adsorbed film that effectively protects the steel from corrosion. These findings align with the practical outcomes, further confirming the potential of **PTA-2** and **PTA-3** molecules as corrosion inhibitors for steel surfaces [85]. Furthermore, table 11 clearly indicates that the adsorption energy values for **PTA-2** and **PTA-3** molecules during the pre-geometry optimization step, i.e. before relaxation (−1686.23 and −1686.33 kcal mol^−1^, respectively), are even more negative than that of **PTA-1** molecule (−1356.17 kcal mol^−1^). This signifies a higher level of protection effectiveness for **PTA-2** and **PTA-3** molecules compared to **PTA-1** molecule. The more negative adsorption energy values for **PTA-2** and **PTA-3** indicate stronger binding to the steel surface, highlighting their superior potential as corrosion inhibitors.

**Table 11.** Data and descriptors computed by the MC simulations for the adsorption of **PTA-1**, **PTA-2** and **PTA-3** molecules on Fe(110).

structures	adsorption energy (kcal mol^−1^)	rigid adsorption energy (kcal mol^−1^)	deformation energy	inhibitor: d*E*_ads_/d*N*_i_	SO_4_^2−^: d*E*_ads_/d*N*_i_	hydronium: d*E*_ads_/d*N*_i_	water: d*E*_ads_/d*N*_i_
**PTA-1**	−1765.78	−1356.17	−409.61	−428.73	−121.33	−82.73	−17.24
**PTA-2**	−2112.34	−1686.23	−426.11	−439.73	−122.11	−81.91	−17.47
**PTA-3**	−2126.36	−1686.33	−440.03	−436.62	−122.02	−81.98	−17.42

The values of d*E*_ads_/d*N*_i_ provide insights into the energy associated with the arrangement of metal and adsorbates (inhibitor molecules), excluding adsorbed water [86]. As shown in table 11, the d*E*_ads_/d*N*_i_ values for both forms of **PTA-2** and **PTA-3** molecules (−439.73 and −436.62 kcal mol^−1^, respectively) are higher than that of **PTA-1** molecule (−428.73 kcal mol^−1^). This indicates a stronger adsorption of **PTA-2** and **PTA-3** molecules compared to **PTA-1** molecule. Additionally, the d*E*_ads_/d*N*_i_ values for water molecules, sulfate ions and hydronium ions are lower than those of **PTA-1**, **PTA-2** and **PTA-3** molecules, suggesting that the inhibitor molecules exhibit robust adsorption compared to water molecules, sulfate ions and hydronium ions. This enhanced adsorption capability of the inhibitor molecules facilitates the displacement of water molecules, hydronium ions and sulfate ions, leading to the formation of a protective adsorbed layer on the steel surface. Consequently, **PTA-1**, **PTA-2** and **PTA-3** molecules effectively adsorb onto the steel surface, forming a robust protective layer that inhibits corrosion in aggressive solutions. This conclusion is supported by both empirical and theoretical investigations.

## 4. Conclusion

Three bis(dimethylpyrazolyl)-aniline-*s*-triazine derivatives were prepared in high yield and characterized to investigate their anticorrosion properties for C-steel.

As the inhibitor concentration increased, the polarization resistance *R*_p_ (*R*_f_ + *R*_ct_) increased, the *C*_dl_ value decreased and the thickness of double layer increased, leading to increase in IE%.

Potentiodynamic polarization studies indicate a sharp decrease in corrosion current and corrosion rate with increase in concentration. The inhibition efficiency of the synthesized inhibitors followed the order **PTA-2** (96.5% at 120 ppm) ≈ **PTA-3** (93.4% at 120 ppm) > **PTA-1** (79.0% at 175 ppm).

The presence of electron-rich group significantly increased the inhibition efficiency of **PTA-2** (−Br) and **PTA-3** (−OCH_3_) compared to **PTA-1** with no functional group.

Comparative studies indicated that both triazine moiety and diazole groups contribute to corrosion inhibition.

The *s*-triazines **PTA-2** and **PTA-3** obey the Langmuir adsorption isotherm, while **PTA-1** obeys the Frumkin model. All the triazines were found to act as mixed-type corrosion inhibitors that reduce iron oxidative dissolution and hinder hydrogen emission reaction.

The values of adsorption equilibrium constant *K°*_ads_ and free energy change Δ*G°*_ads_ revealed that the adsorption of inhibitor onto steel surface was favoured. A corrosion inhibition mechanism proposed the presence of physical and chemical interactions between the *s*-triazine inhibitors and C-steel.

A detailed computational study has provided an insight about the structural features of the molecules and calculation of quantum chemical descriptors such as ionization energy, electron affinity, electronegativity, electronic potential, global softness, global hardness and fraction of electron transfer enabled corroboration of the experimental results.

The values of the energy associated with the metal/adsorbate arrangement d*E*_ads_/d*N*_i_ were calculated by MC simulations. They were almost equal for inhibitor **PTA-2** and **PTA-3** with electron donor groups –Br and –OMe, but higher than that of inhibitor **PTA-1**. This is in analogy with the obtained experimental anticorrosion results obtained for the inhibitors.

## Data Availability

All data are available in the main text or the electronic supplementary material [87]. Samples of novel synthesized compounds are available from the authors.
